# Clinical effect evaluation of postoperative complications of papillary thyroid carcinoma based on individualized intervention and prognostic analysis of precision nursing intervention

**DOI:** 10.3389/fsurg.2026.1761954

**Published:** 2026-07-07

**Authors:** Yu Sun, Gang Pu

**Affiliations:** 1 General Surgery Department, Beihua University Affiliated Hospital, Jilin, Jilin Province, China; 2 Thyroid Surgery Department, Beihua University Affiliated Hospital, Jilin, Jilin Province, China

**Keywords:** individualized intervention, papillary thyroid carcinoma, postoperative complications, precision nursing, prognostic analysis

## Abstract

**Objective:**

This study aims to explore the preventive effects of individualized interventions on postoperative complications in patients with papillary thyroid carcinoma and their impact on patient prognosis.

**Methods:**

A retrospective analysis was conducted, including 1,595 patients with papillary thyroid carcinoma who underwent radical thyroidectomy. Based on the systemic reform of nursing protocols implemented at the end of 2022, the patients were divided into a study group (SG, *n* = 798) and a control group (CG, *n* = 797) according to differences in nursing measures. The CG received routine care, while the SG received individualized interventions in a multidisciplinary perioperative nursing protocol based on routine care, including preoperative thyroid function assessment, intraoperative nerve monitoring and parathyroid protection, and postoperative continuous calcium monitoring.

**Results:**

The overall incidence of postoperative complications in the SG was significantly lower than that in the CG (*P* < 0.001). The incidence of transient recurrent laryngeal nerve injury (*P* = 0.010) and transient hypocalcemia (*P* = 0.007) in the SG were significantly lower than those in the CG. Patients in the SG had lower VAS pain scores at 24 and 72 h postoperatively, and achieved earlier ambulation, feeding, and extubation (*P* < 0.05). The SG had shorter voice recovery time and hospital stay, and higher swallowing function scores (*P* < 0.05). At the 6-month follow-up, patients in the SG had significantly higher scores than those in the CG in terms of thyroid symptoms, mental health, social life, and overall quality of life (*P* < 0.05). The results of the 1-year follow-up showed that the SG had a higher rate of complete voice recovery (*P* = 0.011) and normal calcium metabolism (*P* = 0.016), as well as a lower rate of readmission related to complications (*P* = 0.012).

**Conclusions:**

The implementation of a multidisciplinary perioperative nursing protocol is associated with a reduced incidence of postoperative complications in patients with papillary thyroid carcinoma. It helps alleviate pain intensity and rehabilitation indicators, promotes physiological function recovery, and improves quality of life.

## Introduction

Papillary thyroid carcinoma (PTC) is the most common pathological type of malignant thyroid tumor, accounting for approximately 80%–85% of all thyroid cancers. Its incidence rate has been increasing year by year worldwide (1). With advances in diagnostic technology and increased health awareness, an increasing number of PTCs are discovered in the early stages, and surgical resection remains the main treatment method (2). Although PTC generally has a good prognosis, with a 5-year survival rate of over 95%, postoperative complications remains an important factor affecting patients' quality of life and functional recovery (the follow-up results of this study suggest that nursing models have limited impact on long-term oncological outcomes, but individualized interventions can significantly improve patients' short-term functional recovery and quality of life) (3). The main complications of thyroid surgery include recurrent laryngeal nerve injury, parathyroid dysfunction, postoperative bleeding, and incision infection. Among them, recurrent laryngeal nerve injury and parathyroid dysfunction are the most common complications that have the greatest impact on patients' quality of life (4). According to statistics, the incidence of transient recurrent laryngeal nerve injury after thyroid surgery is 3%–8%, and that of permanent injury is 0.3%–3%. The incidence of transient hypocalcemia is 10%–50%, and that of permanent hypocalcemia is 0.5%–4% (5). These complications not only prolong hospital stay and increase medical expenses, but more importantly, seriously affect the patient's quality of life and social functioning (6).

Traditional nursing models are mainly focused on disease treatment, and the nursing measures are relatively standardized and programmed, lacking in specificity and individualization, and unable to meet the differentiated nursing needs of different patients (7). In recent years, the concept of multidisciplinary perioperative nursing protocol (individualized intervention) has gradually gained attention, emphasizing the development of targeted nursing plans based on the specific conditions of patients. Through precise assessment, dynamic monitoring, and timely intervention, it minimizes the occurrence of complications and promotes patient recovery (8). Individualized intervention is a patient-centered nursing model that develops personalized nursing plans and implements precise nursing measures by comprehensively assessing the patient's condition, surgical method, and personal characteristics (9). This nursing model not only focuses on the disease itself, but also emphasizes the overall needs of patients, including physical, psychological, social, and other dimensions. Studies have shown that individualized nursing intervention has demonstrated good clinical outcomes in multiple surgical fields (10). However, there are relatively few studies on its application in postoperative care of PTC, and its specific impact on postoperative complications and prognosis requires further verification.

Based on the above background, this study retrospectively analyzed the clinical data of 1,595 patients with PTC admitted to our hospital from 2019 to 2024, comparing the efficacy differences between multidisciplinary perioperative nursing protocol (including individualized interventions) and routine care. The purpose was to evaluate the preventive effect of the precision nursing protocols based on individualized interventions on postoperative complications in PTC and their impact on patient prognosis, so as to provide a scientific basis for clinical nursing practice.

## Materials and methods

### Study design

This retrospective cohort study was reviewed and approved by the ethics committee of Beihua University Affiliated Hospital (No. 20260088). The study was conducted in accordance with the relevant requirements of the Declaration of Helsinki. As this was a retrospective analysis, the requirement for informed consent from patients was waived. Based on the systemic reform of nursing protocols implemented at the end of 2022, patients admitted between January 2019 and December 2022 were included in the control group (CG, receiving routine care), while patients admitted between January 2023 and December 2024 were included in the study group (SG, receiving a multidisciplinary, individualized perioperative intervention protocol). All surgeries were performed by the same surgical team, and neuromonitoring technology was fully implemented at our hospital in early 2023.

### Study participants and inclusion and exclusion criteria

Patients with PTC who were treated in the department of thyroid surgery at our hospital between January 2019 and December 2024 were selected as study participants. Cases were screened using the hospital's information system according to the following inclusion and exclusion criteria.

Inclusion criteria: (1) age ≥ 18 years; (2) preoperative fine-needle aspiration or intraoperative frozen section pathology confirmed as PTC; (3) first-time recipient of thyroid surgery; (4) normal thyroid function prior to surgery; (5) complete medical records, including preoperative assessment, intraoperative records, and postoperative care records; (6) postoperative follow-up period ≥12 months.

Exclusion criteria: (1) concurrent malignant tumors; (2) history of neck surgery or radiotherapy; (3) preoperative vocal cord paralysis or parathyroid dysfunction; (4) Concurrent severe dysfunction of vital organs such as the heart, liver, and kidneys; (5) pregnant or lactating women; (6) mental illness or cognitive impairment preventing cooperation with assessment; (7) incomplete medical records or loss to follow-up.

### Grouping method

Patients who met the inclusion criteria were divided into two groups based on the type of nursing intervention:

Patients who received routine care were classified into the CG (*n* = 797). The specific nursing protocol was as follows: (1) preoperative measures included routine hematology testing, assessment of vital signs, and standard preoperative fasting instructions; laryngoscopy and systematic assessment of calcium and phosphorus metabolism were not routinely performed; (2) intraoperative measures included routine positioning and monitoring of vital signs; intraoperative neuromonitoring was not used; (3) postoperative measures included monitoring of vital signs, administration of treatment as prescribed, routine dressing changes, on-demand analgesia (no systematic analgesia protocol), basic life care, and routine health education; continuous serum calcium monitoring and systematic early rehabilitation guidance were not implemented.

Patients who received individualized intervention in the multidisciplinary perioperative nursing protocol were classified into the SG (*n* = 798). Individualized precision nursing protocols were performed based on routine care. Specific measures included: (1) preoperative evaluation and preparation: comprehensive assessment of patients' thyroid function, vocal cord function, and calcium-phosphorus metabolism; development of individualized surgical care plans based on tumor size, location, and lymph node metastasis; psychological assessment and intervention to alleviate preoperative anxiety. (2) Intraoperative protective measures: application of neuromonitoring technology to protect the recurrent laryngeal nerve; fine anatomical techniques to identify and protect the parathyroid glands; *in situ* protection of the parathyroid glands was defined as the preservation of the blood supply to the parathyroid glands and maintenance of their *in situ* position through meticulous dissection under microscopic or loupe magnification during surgery, with this procedure clearly documented in the surgical record; autofluorescence technology was not used in this study; adjustment of body position according to the scope of surgery to reduce nerve traction. (3) Postoperative monitoring and management: continuous monitoring of serum calcium levels for early identification of hypocalcemia; dynamic assessment of vocal function (voice recovery time was defined as the time required for a patient to report that their voice had returned to normal and for laryngoscopic examination to confirm that vocal cord function had returned to normal levels); individualized analgesia plan; drainage management; incision care. (4) Rehabilitation guidance: early functional exercise, including neck mobility and swallowing training; nutritional support plan; psychological support; discharge guidance and follow-up management. Surgeries in both groups were performed by the same surgical team. There were no statistically significant differences in surgical methods or scope between the groups (*P* > 0.05), indicating good comparability.

### Outcome measures

#### Baseline data

General patient information was collected, including age, sex, BMI, tumor diameter, TNM staging, surgical method, and lymph node dissection.

#### Primary outcome measures

(1) Incidence of postoperative complications: including recurrent laryngeal nerve injury (transient/permanent), parathyroid dysfunction (transient/permanent hypocalcemia), postoperative bleeding, incision infection, subcutaneous effusion/hematoma, etc. Transient recurrent laryngeal nerve injury is defined as normal voice recovery within 6 months postoperatively, while permanent injury is defined as failure to recover after 6 months (11). Transient hypocalcemia is defined as serum calcium <2.0 mmol/L postoperatively but returning to normal within 6 months, while permanent hypocalcemia is defined as requiring long-term calcium supplementation (12). Total serum calcium was measured on an empty stomach early in the morning on days 1 and 3 postoperatively, and corrected for albumin levels {correction formula: corrected calcium = measured calcium + 0.8 × [4 − albumin (g/dL)]}. Symptomatic hypocalcemia was defined as a condition in which corrected serum calcium was <2.0 mmol/L, accompanied by clinical symptoms such as numbness in the hands and feet, muscle cramps, or a positive Chvostek's or Trousseau's sign. Biochemical hypocalcemia only involved a decrease in serum calcium without clinical symptoms. Both groups were monitored for serum calcium according to a unified protocol. The SG was given preventive oral calcium carbonate, while the CG was supplemented as needed based on symptoms and serum calcium levels. (2) Implementation and effectiveness evaluation of individualized intervention measures.

#### Secondary outcome measures

(1) Pain assessment: The visual analog scale (VAS) was used to assess postoperative pain intensity. (2) Rehabilitation indicators: Time to ambulation, time of eating, time to extubation, time to voice recovery, and length of hospital stay. (3) Physiological function: Swallowing function score. (4) Quality of life: The Thyroid-Specific Patient-Reported Outcome Measure (ThyPRO) was used to assess the quality of life at 6 months postoperatively. The ThyPRO uses a standard positive scoring system, with a score range of 0–100, where higher scores indicate better quality of life. (5) Prognostic indicators at 1-year follow-up: Recurrence and metastasis, functional recovery, readmission rate, and thyroglobulin level.

### Quality control

All medical staff involved in the study received standardized training. Data collection was performed with standardized scales. Data entry was double-checked by two independent researchers. Regular quality control was conducted.

### Statistical methods

Data analysis was conducted using SPSS 26.0 statistical software. Continuous data were tested for normal distribution and expressed as mean ± standard deviation. Intergroup comparisons were performed using the independent sample *t*-test. Categorical data were expressed as number of cases and percentages. Intergroup comparisons were performed using the chi-square test. Multivariable logistic regression was used to identify independent risk factors for postoperative complications. A *P*-value <0.05 was considered statistically significant.

## Results

### Comparison of baseline data between the two groups

A total of 1,595 patients with PTC were included in this study, including 797 who received routine care (CG) and 798 who received individualized intervention (SG). The baseline characteristics, including age, sex, BMI, and tumor stage, were compared between the two groups. No statistically significant differences were observed in these baseline characteristics between the two groups (*P* > 0.05), indicating good comparability (Table 1).

**Table 1:** Comparison of baseline data between the two groups (mean ± SD)/[*n* (%)].

Baseline data	SG (*n* = 798)	CG (*n* = 797)	*t*/*χ*^2^	*P*
Age (years)	45.28 ± 12.76	46.13 ± 13.24	1.234	0.218
Male/Female	201/597	195/602	0.287	0.592
BMI (kg/m^2^)	23.82 ± 3.19	24.07 ± 3.43	1.826	0.068
Diameter of tumor (cm)	1.83 ± 0.91	1.92 ± 0.98	2.088	0.037
TNM staging			3.421	0.331
Stage I	512 (64.16)	498 (62.48)		
Stage II	186 (23.31)	201 (25.22)		
Stage III	78 (9.77)	75 (9.41)		
Stage IV	22 (2.76)	23 (2.89)		
Surgical method			0.892	0.345
Unilateral resection	485 (60.78)	495 (62.11)		
Bilateral resection	313 (39.22)	302 (37.89)		
Lymph node dissection	623 (78.07)	618 (77.54)	0.068	0.795

### Comparison of postoperative complication rates

The overall postoperative complication rate was significantly lower in the SG compared with the CG (*P* < 0.001). Regarding specific complication types, the SG showed significant advantages in terms of recurrent laryngeal nerve injury and parathyroid dysfunction, with the incidence of transient complications being significantly lower than in the CG (*P* < 0.05). While the difference in the incidence of permanent complications between the two groups was not statistically significant (*P* > 0.05), a downward trend was observed. Other complications, due to their lower incidence, did not differ statistically between the two groups (*P* > 0.05), but the SG also showed a downward trend (Table 2).

**Table 2:** Comparison of postoperative complication rates between the two groups [n (%)].

Types of complications		SG (*n* = 798)	CG (*n* = 797)	*t*/*χ*²	*P*
Overall incidence		76 (9.52)	131 (16.44)	16.872	<0.001
Recurrent laryngeal nerve injury	Transient	12 (1.50)	28 (3.51)	6.591	0.010
	Permanent	1 (0.13)	4 (0.50)	2.137	0.144
Parathyroid dysfunction	Transient hypocalcemia	21 (2.63)	42 (5.27)	7.369	0.007
	Permanent hypocalcemia	2 (0.25)	5 (0.63)	1.124	0.289
Postoperative bleeding		3 (0.38)	8 (1.00)	2.373	0.123
Incision infection		2 (0.25)	7 (0.88)	2.687	0.101
Subcutaneous effusion/hematoma		5 (0.63)	13 (1.63)	3.712	0.054
Other complications		3 (0.38)	6 (0.75)	0.984	0.321

### Implementation and effectiveness evaluation of individualized intervention measures

The implementation rate of each individualized intervention measure in the SG exceeded 85%, with 100% implementation of preoperative thyroid function assessment, fine dissection techniques, continuous calcium monitoring, and early functional assessment. Comparison of complication rates between patients with and without specific interventions suggested that preoperative assessment, intraoperative protection, and postoperative monitoring were all associated with a reduction in the incidence of corresponding complications. In particular, the use of neuromonitoring was associated with a decreased rate of recurrent laryngeal nerve injury (1.16% vs. 4.59%, *P* = 0.009). However, as neuromonitoring was fully implemented at our hospital in early 2023, this association may be partly influenced by temporal factors. Parathyroid *in situ* protection was also linked to a lower incidence of hypocalcemia (2.53% vs. 5.81%, *P* = 0.077) (Table 3).

**Table 3:** Implementation and effectiveness evaluation of individualized intervention measures.

Intervention measures	Implementation rate	Complication rate	Complication rate in non-implementation group	*χ* ^2^	*P*
Preoperative assessment and preparation					
Thyroid function assessment	798 (100.00)	12/798 (1.50)	28/797 (3.51)	6.591	0.010
Laryngoscopic examination	756 (94.74)	10/756 (1.32)	3/42 (7.14)	7.842	0.005
Calcium-phosphorus metabolism assessment	785 (98.37)	20/785 (2.55)	3/13 (23.08)	15.287	<0.001
Intraoperative protective measures					
Neuromonitoring	689 (86.34)	8/689 (1.16)	5/109 (4.59)	6.834	0.009
Fine dissection techniques	798 (100.00)	13/798 (1.63)			
Parathyroid *in situ* protection	712 (89.22)	18/712 (2.53)	5/86 (5.81)	3.126	0.077
Postoperative monitoring					
Continuous calcium monitoring	798 (100.00)	23/798 (2.88)	47/797 (5.90)	8.432	0.004
Early functional assessment	798 (100.00)	76/798 (9.52)	131/797 (16.44)	16.872	<0.001

### Differences in pain intensity and recovery indicators between the two groups

**Figure 1:**
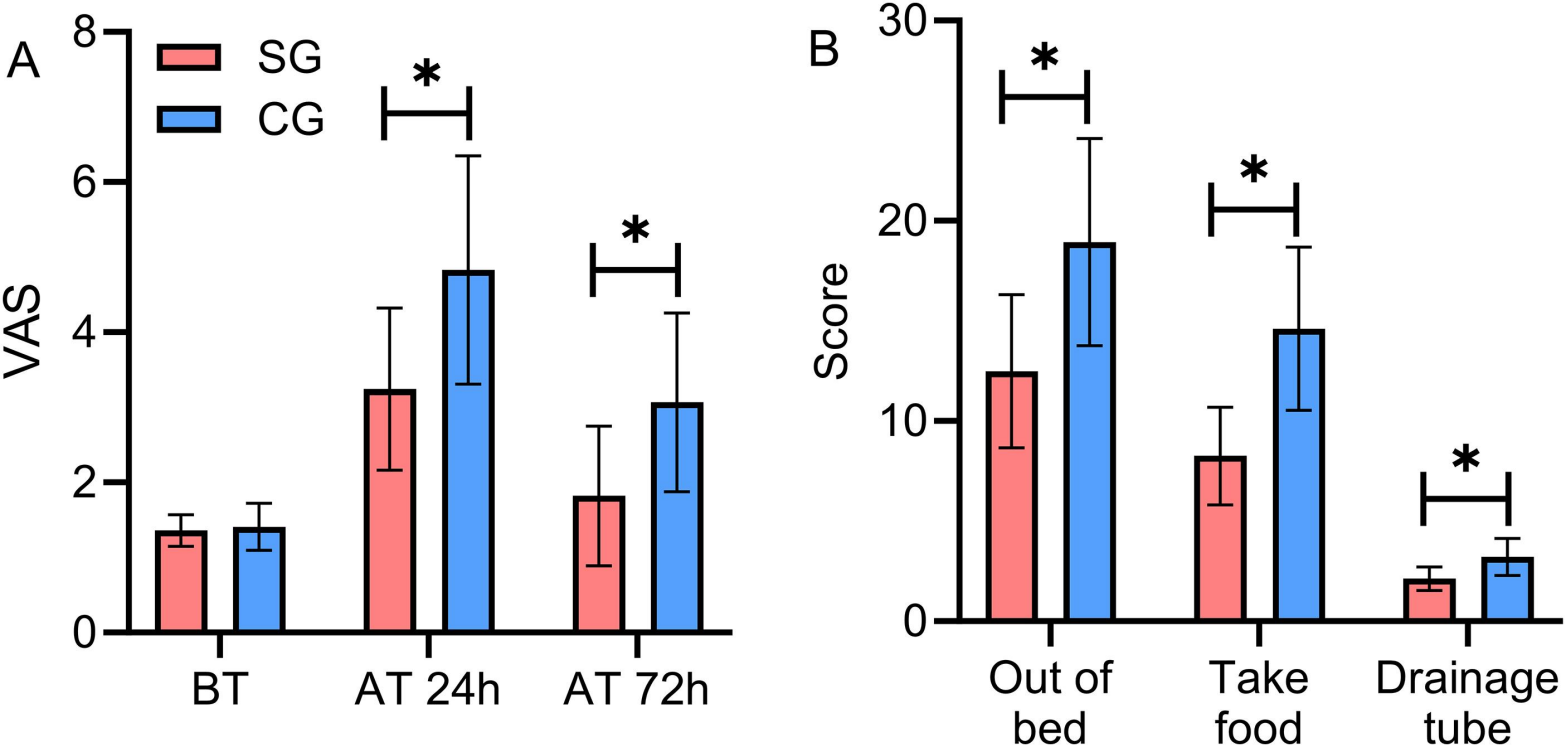
Differences in pain intensity and recovery indicators between the two groups. Patients in the SG had lower VAS pain scores at 24 and 72 h postoperatively (*P* < 0.05) **(A)**. Patients in the SG achieved earlier ambulation, feeding, and extubation (*P* < 0.05) **(B)**. BT, before treatment; AT24h, 24 h after treatment; AT72h, 72 h after treatment. *Indicates statistically significant differences between groups.

There was no statistically significant difference in VAS scores between the two groups before surgery (*P* > 0.05). However, VAS scores in the SG were significantly lower than those in the CG at 24 and 72 h after surgery (*P* < 0.05). Furthermore, patients in the SG achieved earlier ambulation, feeding, and extubation than those in the CG (*P* < 0.05) (Figure 1).

### Differences in physiological function recovery indicators between the two groups

**Figure 2:**
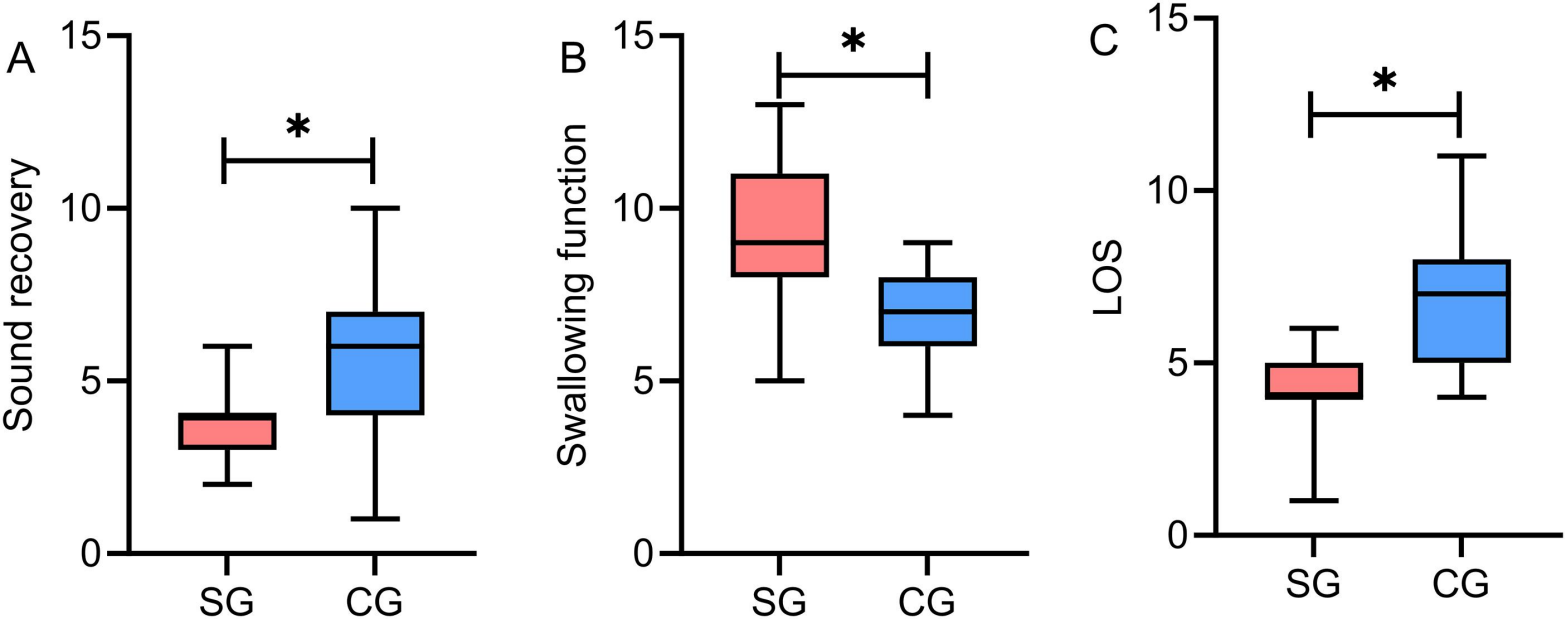
Differences in physiological function recovery indicators between the two groups. The voice recovery time **(A)** and hospital stay **(C)** of patients in the SG were shorter than those in the CG, and the swallowing function score **(B)** was higher than that in the CG (*P* < 0.05). *Indicates statistically significant differences between groups.

The voice recovery time and hospital stay of patients in the SG were shorter than those in the CG, and the swallowing function score was higher than that in the CG (*P* < 0.05) (Figure 2).

### Comparison of quality of life scores between the two groups at 6 months postoperatively

**Figure 3:**
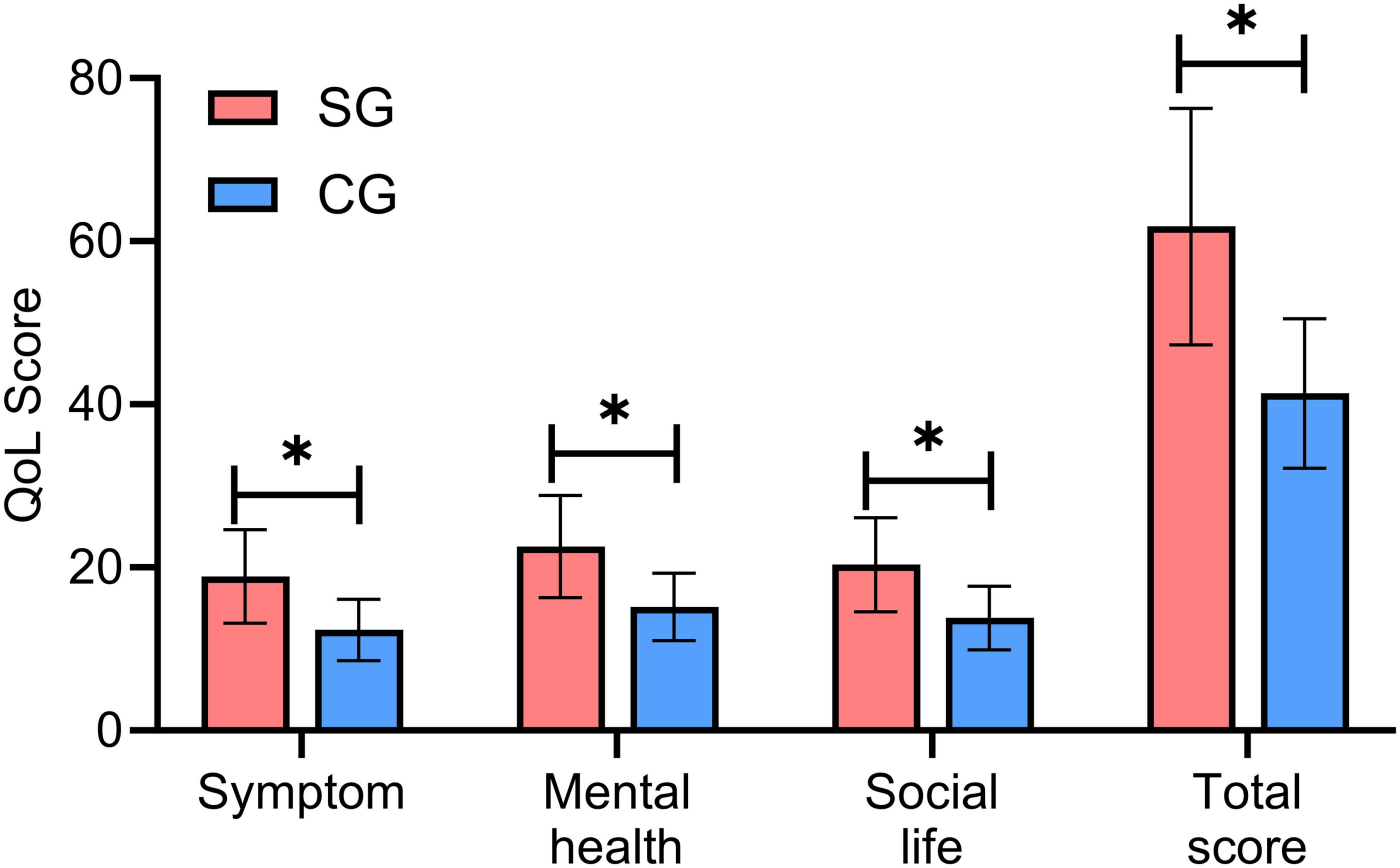
Comparison of quality of life scores between the two groups at 6 months postoperatively. The SG showed significantly higher scores in thyroid symptoms, mental health, social life, and total scores than the CG (*P* < 0.05). *Indicates statistically significant differences between groups.

Follow-up data were collected 6 months after surgery. Using the Thyroid-Specific QoL Scale, the SG showed significantly higher scores in thyroid symptoms, mental health, social life, and total scores than the CG (*P* < 0.05) (Figure 3).

### Comparison of prognostic indicators between the two groups at one-year follow-up

**Figure 4:**
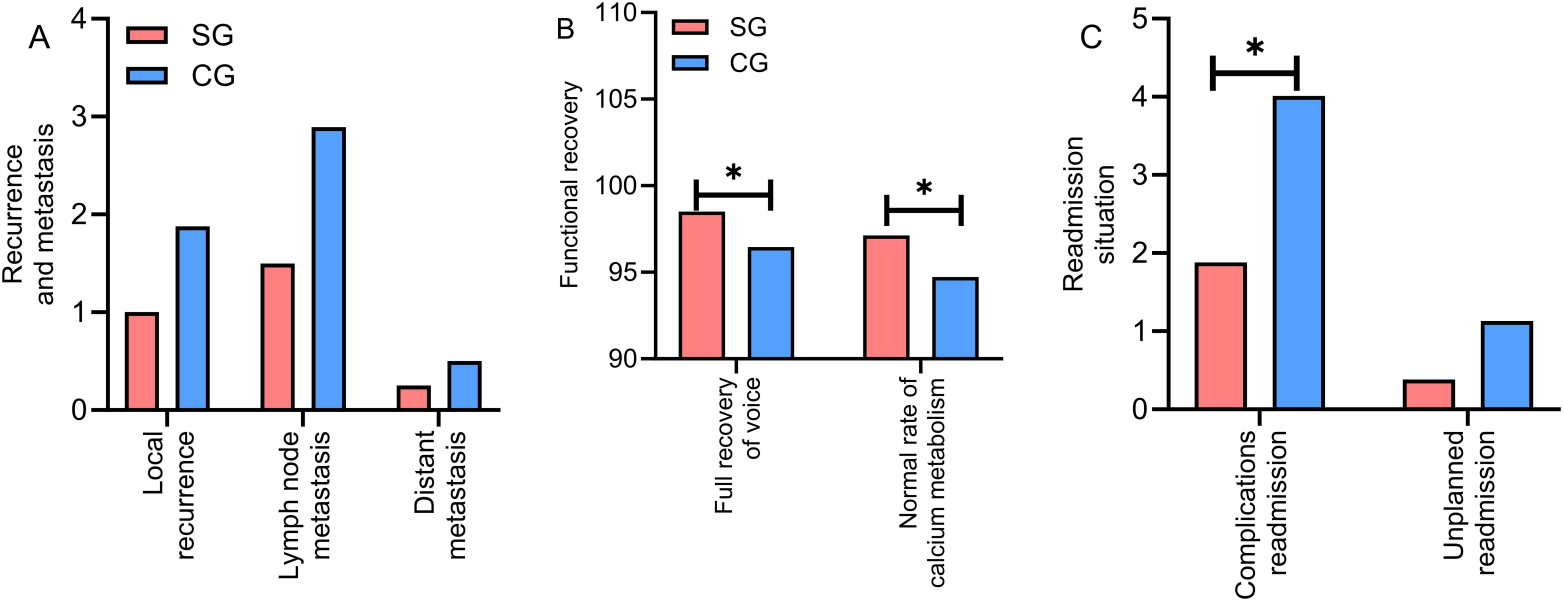
Comparison of prognostic indicators between the two groups at one-year follow-up. Individualized nursing measures helped improve the rate of complete voice recovery and normal calcium metabolism (*P* < 0.05). *Indicates statistically significant differences between groups.

The one-year follow-up results showed no statistically significant difference in tumor recurrence and metastasis between the two groups (*P* > 0.05). This suggests that the nursing model has a limited impact on oncological prognosis. However, in terms of functional recovery, individualized nursing measures helped improve the rate of complete voice recovery and normal calcium metabolism (*P* < 0.05) (Figure 4).

### Analysis of risk factors for postoperative complications

Univariable analysis of risk factors for postoperative complications was performed, with the presence of postoperative complications as the dependent variable, and sex, age, surgical method, and the presence of individualized nursing as independent variables. The results indicated that TNM staging, surgical method, sex, lymph node dissection, individualized nursing, age, BMI, and tumor diameter were all associated with postoperative complications (*P* < 0.1) (Table 4). Further multivariable logistic regression analysis revealed that age ≥50 years, BMI ≥ 25 kg/m^2^, tumor diameter ≥ 2 cm, TNM stage III-IV, lymph node dissection, and the absence of individualized nursing were independent risk factors for postoperative complications (*P* < 0.05) (Table 5).

**Table 4:** Univariable analysis of risk factors for postoperative complications.

Prognostic indicators		Complications (*n* = 207)	No complications (*n* = 1,388)	*t*/*χ*^2^	*P*
TNM staging	I	112 (54.11%)	898 (64.73%)	19.234	<0.001
	II	56 (27.05%)	331 (23.85%)		
	III	28 (13.53%)	125 (9.01%)		
	IV	11 (5.31%)	34 (2.45%)		
Surgical method	Unilateral resection	107 (51.69%)	873 (62.90%)	12.658	<0.001
	Bilateral resection	100 (48.31%)	515 (37.10%)		
Sex	Male	61 (29.47%)	335 (24.14%)	2.847	0.092
	Female	146 (70.53%)	1,053 (75.86%)		
Lymph node dissection	Yes	176 (85.02%)	1,065 (76.73%)	8.924	0.003
	No	31 (14.98%)	323 (23.27%)		
Individualized nursing	Yes	76 (36.71%)	722 (52.02%)	16.872	<0.001
	No	131 (63.29%)	666 (47.98%)		
Age (years)		48.92 ± 13.56	45.14 ± 12.87	3.912	<0.001
BMI (kg/m^2^)		24.78 ± 3.62	23.86 ± 3.27	3.689	<0.001
Tumor diameter (cm)		2.31 ± 1.12	1.82 ± 0.93	6.845	<0.001

**Table 5:** Multivariable logistic regression analysis of risk factors for postoperative complications.

Risk factors	*B*	S.E	Wald	*P*	OR	95% CI
Age ≥ 50 years	0.532	0.168	10.082	0.001	1.703	1.226–2.365
BMI ≥ 25 kg/m^2^	0.389	0.172	5.134	0.023	1.476	1.054–2.067
Tumor diameter ≥ 2 cm	0.825	0.181	20.745	<0.001	2.282	1.600–3.256
TNM stage III-IV	0.678	0.213	10.143	0.001	1.970	1.297–2.991
Bilateral resection	0.472	0.176	7.198	0.007	1.603	1.135–2.264
Lymph node dissection	0.396	0.198	3.999	0.046	1.486	1.008–2.191
Absence of individualized nursing	0.744	0.169	19.376	<0.001	2.104	1.510–2.932

## Discussion

### Effect of individualized intervention on postoperative complications

In this study, the overall incidence of postoperative complications was 9.52% in the SG (receiving individualized interventions in the multidisciplinary perioperative nursing protocol), which was significantly lower than 16.44% observed in the CG (receiving routine care) (*P* < 0.001). These findings highlight the potential role of individualized nursing interventions in mitigating postoperative complications for PTC. This result is generally consistent with the results of relevant studies both domestically and internationally (13, 14). After a specific analysis of various complications, we found that individualized intervention was particularly effective in preventing recurrent laryngeal nerve injury and parathyroid dysfunction. In the SG, the incidence of transient recurrent laryngeal nerve injury was 1.50%, a 57% reduction compared with 3.51% in the CG (*P* = 0.010). This difference was associated with the use of intraoperative neuromonitoring (1.16% in the implementation group vs. 4.59% in the non-implementation group, *P* = 0.009). Neuromonitoring not only accurately locates the course of nerves, but also assesses the functional status of nerves in real time, which is associated with timely intraoperative adjustments and the prevention of nerve damage (15). Notably, neuromonitoring was fully adopted at our hospital in early 2023, coinciding with the enrollment period of the SG. Therefore, the observed association may be partially influenced by the surgical learning curve or temporal factors, and causal inferences should be interpreted with caution. In terms of parathyroid protection, individualized intervention also showed obvious advantages. The incidence of transient hypocalcemia in the SG was 2.63%, significantly lower than 5.27% in the CG (*P* = 0.007). Although the difference did not reach statistical significance, parathyroid *in situ* protection technology was associated with a reduction in hypocalcemia incidence (2.53% vs. 5.81%, *P* = 0.077). Based on the literature, the fine anatomical technique and intraoperative parathyroid gland identification and protection play an important role in reducing postoperative hypocalcemia (16).

### Implementation effectiveness of individualized interventions

This study conducted a detailed analysis of the implementation and effectiveness of various individualized interventions. The results showed that the implementation rate of each measure in the SG exceeded 85%, with a 100% implementation rate for preoperative thyroid function assessment, fine dissection techniques, continuous calcium monitoring, and early functional assessment. This high implementation rate ensures the effective implementation of the individualized care plan. In addition, the results of the study also suggest that preoperative assessment is of great significance. For example, the implementation of laryngoscopy was associated with a reduced rate of recurrent laryngeal nerve injury (1.32% vs. 7.14%, *P* = 0.005), and the calcium-phosphorus metabolism assessment was linked to a lower incidence of hypocalcemia (2.55% vs. 23.08%, *P* < 0.001). The above data strongly suggest the potential value of adequate preoperative assessment in preventing postoperative complications (17). Notably, the incidence of hypocalcemia observed in this study was lower than that reported in previous studies, which may be attributable to the proactive preventive management and optimized monitoring methods applied in the SG. Furthermore, biochemical hypocalcemia was defined using a strict criterion (corrected serum calcium < 2.0 mmol/L), and therefore some cases may have been underdetected.

### Effect of individualized intervention on rehabilitation quality

In addition to its association with lower complication rates, individualized interventions were also linked to improvements in patient rehabilitation quality. VAS pain scores showed that the pain intensity in the SG was significantly lower than that in the CG at 24 and 72 h postoperatively (*P* < 0.05), which is similar to the results of other studies (18). This study suggests that postoperative pain significantly reduces the surgical experience of thyroid surgery patients and has a negative impact on their postoperative recovery. Effective pain control may improve patient comfort during the perioperative period and promote early mobilization and functional recovery. In terms of functional recovery, the times to ambulation, eating, and extubation in the SG was significantly shorter than that in the CG (*P* < 0.05). In particular, the shortening of voice recovery time and hospital stay directly reflected the positive association of individualized care on accelerated recovery. The improvement of the above indicators is closely related to the implementation of measures such as early functional exercise and individualized nutritional support (19, 20). Further quality of life assessments confirmed the long-term benefits of individualized intervention. Six months after surgery, the scores of patients in the SG were significantly better than those in the CG in various dimensions such as thyroid symptoms, mental health, and social life. This result indicates that the multidisciplinary perioperative nursing protocol is associated not only with improved short-term clinical outcomes but also with enhanced long-term quality of life for patients (21, 22).

### Prognosis and risk factor analysis

One-year follow-up revealed that while there were no significant differences between the two groups in terms of tumor recurrence and metastasis, the SG demonstrated superior functional recovery, with a complete voice recovery rate of 98.50% and a normal calcium metabolism rate of 97.12%, both significantly higher than those in the CG (*P* < 0.05). In addition, the complication-related readmission rate in the SG was only 1.88%, significantly lower than 4.01% in the CG (*P* = 0.012), suggesting the long-term benefits of individualized care. Multivariable logistic regression analysis showed that the absence of individualized care was associated with an increased risk for postoperative complications (OR = 2.104, *P* < 0.001), further confirming the protective effect of individualized care. Meanwhile, factors such as age ≥50 years, BMI ≥ 25 kg/m^2^, tumor diameter ≥ 2 cm, TNM stage III-IV, and lymph node dissection were also independent risk factors for complications. The above results have also been mentioned in other studies (23, 24). The present study suggests that the discovery of the above risk factors may help medical workers to differentiate and manage patients undergoing thyroid cancer surgery and assist in formulating more accurate individualized care plans.

### Clinical significance, limitations, and future prospects of the study

The results of this study have important clinical implications. The multidisciplinary perioperative nursing protocol, through precise assessment, targeted prevention, and dynamic monitoring, is associated with a reduction in postoperative complications, improved quality of recovery, and favorable long-term prognosis in patients with PTC. The successful implementation of this care model requires the collaboration of a multidisciplinary team, including surgeons, anesthesiologists, nurses, rehabilitation therapists, etc. This study also has certain limitations. First, patients were grouped according to the timing of nursing protocol changes. Consequently, the effects of the surgical learning curve, technical improvements, or other temporal factors on the results cannot be fully ruled out. Second, neuromonitoring was not applied to all patients in the SG (implementation rate 86.34%), making it difficult to completely separate its independent effect on complications from that of nursing interventions. The potential synergistic effect of the two needs to be further clarified in future prospective studies. Third, due to the retrospective design of the study, preoperative baseline ThyPRO scores were not available; therefore, the postoperative differences in quality of life should be interpreted with consideration of baseline status. Fourth, the results of a single-center study have limited generalizability, and the follow-up period is relatively short; the longer-term effects remain to be observed. In the future, multicenter, prospective, randomized controlled studies should be conducted to further verify the effectiveness of multidisciplinary perioperative nursing protocol and explore more optimized care plans.

## Conclusion

The implementation of a multidisciplinary perioperative nursing protocol (including individualized interventions) is associated with reduced postoperative complication rates, shorter recovery time, and improved quality of life in patients with PTC, demonstrating significant clinical value. It is recommended to promote the application of individualized nursing models in clinical practice, especially for high-risk patients, who should be given more precise and comprehensive nursing interventions in order to achieve optimal clinical outcomes. Prospective randomized controlled trials are needed in the future to provide a higher level of evidence.

## Data Availability

The original contributions presented in the study are included in the article/Supplementary Material, further inquiries can be directed to the corresponding author.
